# Extreme Drug Tolerance of *Mycobacterium abscessus* “Persisters”

**DOI:** 10.3389/fmicb.2020.00359

**Published:** 2020-03-04

**Authors:** Yee-Kuen Yam, Nadine Alvarez, Mei-Lin Go, Thomas Dick

**Affiliations:** ^1^Center for Discovery and Innovation, Hackensack Meridian Health, Nutley, NJ, United States; ^2^Public Health Research Institute, New Jersey Medical School, Rutgers, The State University of New Jersey, Newark, NJ, United States; ^3^Department of Pharmacy, Faculty of Science, National University of Singapore, Singapore, Singapore; ^4^Department of Medical Sciences, Hackensack Meridian School of Medicine at Seton Hall University, Nutley, NJ, United States

**Keywords:** NTM, drug tolerance, biofilm, nutrient starvation, oxygen starvation

## Abstract

Persistence of infection despite extensive chemotherapy with antibiotics displaying low MICs is a hallmark of lung disease caused by *Mycobacterium abscessus* (Mab). Thus, the classical MIC assay is a poor predictor of clinical outcome. Discovery of more efficacious antibiotics requires more predictive *in vitro* potency assays. As a mycobacterium, Mab is an obligate aerobe and a chemo-organo-heterotroph – it requires oxygen and organic carbon sources for growth. However, bacteria growing in patients can encounter micro-environmental conditions that are different from aerated nutrient-rich broth used to grow planktonic cultures for MIC assays. These *in vivo* conditions may include oxygen and nutrient limitation which should arrest growth. Furthermore, Mab was shown to grow as biofilms *in vivo*. Here, we show Mab Bamboo, a clinical isolate we use for Mab drug discovery, can survive oxygen deprivation and nutrient starvation for extended periods of time in non-replicating states and developed an *in vitro* model where the bacterium grows as biofilm. Using these culture models, we show that non-replicating or biofilm-growing bacteria display tolerance to clinically used anti-Mab antibiotics, consistent with the observed persistence of infection in patients. To demonstrate the utility of the developed “persister” assays for drug discovery, we determined the effect of novel agents targeting membrane functions against these physiological forms of the bacterium and find that these compounds show “anti-persister” activity. In conclusion, we developed *in vitro* “persister” assays to fill an assay gap in Mab drug discovery compound progression and to enable identification of novel lead compounds showing “anti-persister” activity.

## Introduction

Non-tuberculous mycobacteria (NTM), mycobacteria other than *Mycobacterium tuberculosis* complex and *Mycobacterium leprae*, are ubiquitous environmental bacteria that can cause difficult-to-cure lung disease (Falkinham, 2013). The disease mostly affects people with pre-existing pulmonary conditions including cystic fibrosis and bronchiectasis. Thus, soil and water dwelling NTM are, in contrast to the obligate parasite *M. tuberculosis*, opportunistic pathogens (Prevots and Marras, 2015). Infections with *Mycobacterium abscessus* (Mab), a fast-growing NTM, are particularly difficult to eradicate (Davidson, 2018; Ryan and Byrd, 2018; Chen et al., 2019). While there is no drug regimen of proven or predictable efficacy for Mab lung disease, a typical treatment consists of a macrolide (e.g., azithromycin or clarithromycin) combined with an aminoglycoside (amikacin) and a beta-lactam (imipenem or cefoxitin). Other drugs, including tetracyclines (minocycline or tigecycline), an oxazolidinone (linezolid), a fluoroquinolone (moxifloxacin), and the riminophenazine clofazimine may also be used (Griffith et al., 2007; Brown-Elliott et al., 2012; Chen et al., 2019). Treatment often goes on for years and negative sputum culture for 1 year – while on therapy – is recommended before a patient is declared cured. Despite this extensive chemotherapy, treatment success rates are low (Griffith et al., 2007). Thus, Mab lung disease can be considered a chronic infection that is rarely cured by our currently used drugs.

More efficacious antibiotics are needed to provide reliable regimens (Daniel-Wayman et al., 2019). We recently summarized status, issues, and gaps around NTM/Mab drug discovery (Falkinham, 2018; Wu et al., 2018). One major issue is the disconnect between MIC (Minimum Inhibitory Concentration) and clinical outcome. Usually for bacterial infections, a low MIC translates into a successful clinical outcome. Typically, anti-Mab antibiotics have attractive MICs. However, in the case of Mab disease low MIC does not translate into rapid cure. Thus, the standard MIC assay is of limited predictive value and more predictive assays are needed as secondary bacteriological potency readouts (Wu et al., 2018).

What may be the reason for the limited predictive value of the standard bacterial MIC assay? Mycobacteria are obligate aerobes, requiring oxygen for growth. Furthermore, they are chemo-organo-heterotrophs – they require organic carbon sources for growth. The MIC of an anti-mycobacterial is the concentration that suppresses growth of a planktonic culture growing in aerated, nutrient-rich broth. The pathology of Mab lung disease suggests that the micro-environmental conditions encountered by the pathogen in its host differ substantially from the *in vitro* culture conditions provided in aerated, nutrient-rich broth (Swenson et al., 2018). *In vivo* micro-environments may include oxygen deprivation and nutrient limitation. These conditions would affect the pathophysiology of the bacteria: *in vivo*, Mab may exist in largely slow- or non-growing states in stark contrast to the rapidly replicating state generated in the standard *in vitro* assay used for traditional drug testing. If the *in vivo* bacteria are not in a state of growth, growth inhibition potency of an antibiotic is not a meaningful pharmacodynamic parameter. Rather, the bactericidal activity of an antibiotic should be used. The Minimum Bactericidal Concentration, the concentration that reduces initial CFU by, for example 90% (MBC_90_), is traditionally again determined for cultures growing rapidly in aerated nutrient-rich broth. For the obligate parasite *M. tuberculosis* and the saprophytic mycobacterial model organism *Mycobacterium smegmatis*, it was shown that these bacteria are capable of surviving anaerobiosis (Wayne, 1976; Dick et al., 1998; Lim et al., 1999) and nutrient starvation (Gengenbacher et al., 2010; Wu et al., 2016) for extended periods of time by adopting a state of “non-replicating persistence” (Boon and Dick, 2012). These non-replicating cultures display drug tolerance or phenotypic (as opposed to genetic) drug resistance: non-growing bacteria are not, or much less, killed by bactericidal antibiotics than growing bacteria. In other words, the MBC_90_ of drugs against non-replicating cultures shift to higher values when compared to MBC_90_ values against standard growing cultures (Lakshminarayana et al., 2015). As most antibiotics target growth-essential functions such as protein and cell wall synthesis, a plausible explanation for the loss of bactericidal activity when shifting from growth to a metabolically quiescent non-replicating state is that the antibiotic targets lose their “essentiality.” Typically, tuberculosis drug discovery flowcharts contain “persister assays” (see Materials and Methods for definition), in addition to the standard MIC and bactericidal assays using aerated nutrient rich cultures (Franzblau et al., 2012). In these “persister assays,” the bactericidal activity of novel leads is determined against non-replicating (for instance anerobic or nutrient-starved) *M. tuberculosis*. Interestingly, Mab was shown to grow in patients not only as single cells but also as biofilm (Qvist et al., 2015; Fennelly et al., 2016). For the saprophytic *M. smegmatis*, it was shown that growing biofilm can show a shift in biofilm MIC, the concentration required to inhibit biofilm growth, when compared to the respective MIC values observed for planktonic cultures (Teng and Dick, 2003). Taken together, Mab growing in patients may adopt a range of physiological states that are phenotypically resistant to the growth inhibitory and/or bactericidal effects that drugs may exert against rapidly growing suspension culture.

Here, we describe the establishment and application of “persister assays” for Mab drug discovery. We chose the strain Mab subspecies *abscessus* Bamboo (Yee et al., 2017), a recent clinical isolate that our lab uses extensively for screening to identify anti-Mab actives (Aziz et al., 2017; Low et al., 2017). First, we show that Mab Bamboo is capable of surviving oxygen deprivation and nutrient starvation for extended periods of time in a non-replicating state and can grow as a biofilm attached to a surface. Profiling of currently used anti-Mab antibiotics in these assays shows that these physiological forms display drug tolerance, consistent with clinically observed persistence of infection (Wu et al., 2018). Finally, we employ these assays to determine activity of novel agents targeting bacterial membrane associated functions and show that these agents display “anti-persister” activity, supporting the utility of these assays.

## Materials and Methods

### Bacterial Strain, Culture Media, and Chemicals

*M. abscessus* Bamboo is a clinical isolate from a patient with amyotrophic lateral sclerosis and bronchiectasis. The strain was provided by Wei Chang Huang (Taichung Veterans General Hospital, Taichung, Taiwan). The strain was previously whole-genome sequenced which showed that it belongs to *M. abscessus* subsp. *abscessus* and harbors an inactive, clarithromycin-sensitive *erm*(41) C28 sequevar (Yee et al., 2017). The strain was maintained in complete Middlebrook 7H9 broth (BD Difco 271310, Spark, MD, United States) supplemented with 0.2% glycerol (Fisher Scientific G31-1, Fair Lawn, NJ, United States), 0.05% Tween-80 (Sigma-Aldrich P1754-1L, St. Louis, MO, United States) and 10% Middlebrook albumin-dextrose-catalase (ADC) (BD BBL 212352, Sparks, MD, United States). CFU were determined using Middlebrook 7H10 agar (BD Difco 262710 Sparks, MD, United States) supplemented with 0.2% glycerol and 10% Middlebrook oleic acid-albumin-dextrose-catalase (OADC) (BD BBL 212351 Sparks, MD, United States). Antibiotics were purchased from Sigma-Aldrich St. Louis, MO, United States (with exception of imipenem which was purchased from Cayman Chemicals, Ann Arbor, MI, United States), dissolved in 100% DMSO (MP Biomedicals 190186, Solon, OH, United States). Amikacin, imipenem, moxifloxacin and cefoxitin were dissolved in water as recommended by manufacturer and sterilized using 0.2 μm PTFE membrane filters (Acrodisc PALL 4225, Ann Arbor, MI, United States) before use. The N-substituted indolemethylamine IMA6 *N*-[(6-Trifluoromethyl-1*H*-indol-2-yl)methyl]cyclooctanamine and the N^1^,N^3^-dialkyl substituted dioxonaphthoimidazolium SA23 1-Butyl-2-methyl-3-octyl-4,9-dioxo-4,9-dihydro-1*H*-naphtho[2,3-*d*]imidazol-3-ium bromide were synthesized in-house in the context of *M. tuberculosis* drug discovery projects (Supplementary Figures S1, S2; Dick and Go, unpublished).

### Growth in Aerated Nutrient-Rich Broth and MIC, MBC_90_ Determination

**FIGURE 1:**
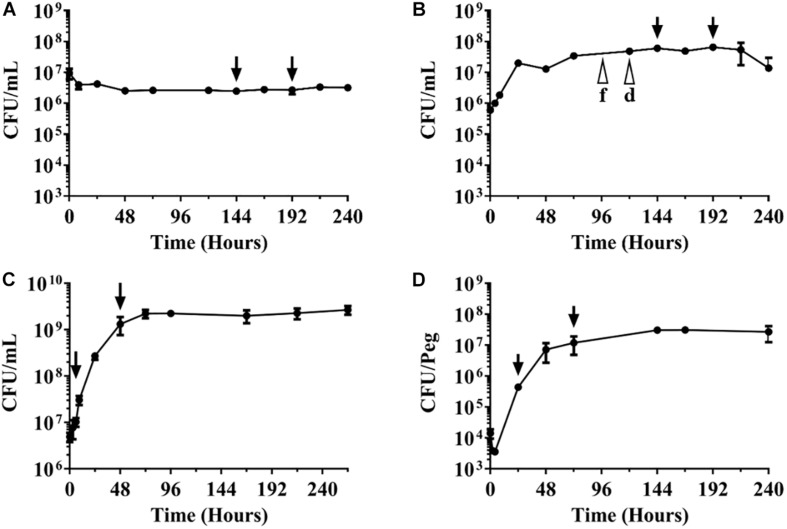
Growth-survival curves of *M. abscessus* Bamboo under various culture conditions. CFU/mL over time are shown. The different culture types were inoculated with exponentially growing aerated nutrient-rich planktonic cultures. **(A)** Survival in phosphate buffered saline devoid of any nutrients. **(B)** Growth and survival in nutrient-rich broth in sealed containers with limited oxygen supply. f, d indicate fading and decolorization of the oxygen indicator methylene blue. **(C)** Growth in aerated nutrient-rich broth. **(D)** Growth as biofilm on plastic surface. Arrows indicate time points when drugs were added and samples taken for CFU enumeration for experiments shown in Table 1. Drug treatment times (48 h) were kept constant across the various culture conditions. The experiments were carried out at least two times independently in duplicate and mean values with standard deviations are shown.

Exponentially growing Mab Bamboo precultures were generated from frozen stocks that were diluted into 12 mL 7H9 medium in 50 mL conical Falcon tubes (Corning 352070, Reynosa, TM, United States). Precultures were aerated with stir-bars at 450 rpm on magnetic stirring platforms at 37°C and grown overnight to mid-log phase (OD_600_ = 0.4–0.6). To grow experimental aerated nutrient rich cultures, mid-log phase precultures were diluted to OD_600_ = 0.05 (4.9 × 10^6^ CFU/mL) and 1 mL aliquots were transferred into 14 mL vented, round-bottom tubes (Thermo Fisher 150268, Rochester, NY, United States). The tubes were incubated on a tilted rack at 37°C on an orbital shaker at 220 rpm. Growth was measured by CFU determination. Serial dilution in phosphate buffered saline (Thermo Fisher 10010023) containing 0.025% Tween-80 (PBS/Tween-80) were plated on 7H10 agar. To determine the MIC of antibiotics, appropriate ranges of drug concentration were added at time point 0 h (Figure 1C) and incubated for 48 h with antibiotic before CFU were determined. The lowest concentration that prevented growth in CFU was defined as the MIC of the respective drug. Determination of MBC_90_, the concentration of drug that reduces viable numbers 10-fold compared to the starting CFU/mL, were carried out the same way with increased concentrations of drug up to 100 μM.

**TABLE 1:** Antibiotic susceptibilities of *M. abscessus* Bamboo under various culture conditions.

					**Nutrient starved**	**Anaerobic**
**Drug**	**Broth**	**Biofilm**	**Broth**	**Biofilm**	**non-replicating**	**non-replicating**
			
	**MIC (μM) ([x], μg/mL)**		**MBC_90_ (μM)**		**MBC_90_ (μM)**	
Clarithromycin	0.2[0.1]	3	>100	>100	>100	>100
Azithromycin	2[1.6]	2	>100	>100	>100	>100
Amikacin	2[1.2]	0.5	12.5	25	>100	>100
Minocycline	31[15.3]	>100	>100	>100	>100	>100
Tigecycline	2[1.2]	9	100	75	>100	>100
Imipenem	5[1.6]	20	12.5	50	>100	>100
Cefoxitin	16[7.2]	50	25	100	>100	>100
Linezolid	4[1.4]	12.5	>100	>100	>100	>100
Moxifloxacin	2[0.9]	6	3	25	>100	>100
Clofazimine	5[2.4]	9	>100	12.5	>100	31
IMA6^#^	3[1.0]	6	3	75	6	38
SA23^#^	3[1.4]	75	25	100	25	9

*Broth MIC, MIC for aerated nutrient-rich planktonic culture (see Figure 1C). Note: this column includes the corresponding MIC values in μg/mL for reference. Biofilm MIC, MIC for Mab growing attached to a plastic surface (see Figure 1D). Broth MBC_90_, MBC_90_ for aerated nutrient-rich planktonic culture (see Figure 1C). Biofilm MBC_90_, MBC_90_ for Mab growing attached to a plastic surface (see Figure 1D). Nutrient-starved, non-replicating MBC_90_, MBC_90_ for non-replicating, nutrient-starved culture (see Figure 1A). Anaerobic, non-replicating culture MBC_90_, MBC_90_ for non-replicating, anerobic culture (see Figure 1B). ^#^IMA6 and SA23 are representatives of MmpL3-targeting N-substituted indolemethylamines (IMA6) and redox-cycling N^1^,N^3^-dialkyl substituted dioxonaphthoimidazoliums (SA23) derived from M. tuberculosis drug discovery projects (see section Materials and Methods, Dick and Go, unpublished, Supplementary Figures S1, S2). The experiments were carried out at least two times independently in duplicate and mean values are shown.*

### Growth as Biofilm and MIC, MBC_90_ Determination

Innovotech MBEC 96-well Biofilm Assay Plates (Innovotech 19111, Edmonton, AB, United States) were used, and the supplier’s manual was followed with minor modifications. Mid-log phase Mab Bamboo precultures (section Growth in Aerated Nutrient-Rich Broth and MIC, MBC_90_ Determination) were spun down at 3200 × *g* for 10 min at 25°C and washed with 7H9 medium without Tween-80 (7H9nt). Bacteria were resuspended into 25 mL 7H9nt to an OD_600_ = 0.0125 (1.0 × 10^6^ CFU/mL). One hundred and fifty microliter of culture was dispensed into each well of MBEC multi-titer plates and the polystyrene protrusions (pegs) of the MBEC lid were inserted into the culture-containing wells for 24 h at 37°C on an orbital shaker at 110 rpm to allow attachment of the bacteria to the pegs and initiation of biofilm growth. The lids with the pegs were transferred to a new MBEC multi-titer plate containing 150 μL of fresh 7H9nt medium per well without bacteria (time point 0 h in Figure 1D). After that, the pegs with growing biofilm were transferred once a day to a new multi-titer plate containing fresh 7H9nt medium. To measure growth of the biofilm formed on the peg, the pegs were washed in 200 μL of 7H9nt medium before they were aseptically removed and placed in 1.7 mL microcentrifuge tubes (VWR 87003-294, Rador, PA, United States) containing 500 μL PBS/Tween-80. The microcentrifuge tubes were vigorously vortexed at 2,000 rpm for 90 s at 25°C to detach the bacteria from the pegs before samples were serially diluted and plated for the determination of CFU/peg. To determine the biofilm MIC of antibiotics, appropriate drug concentrations were added at the 24 h timepoint (Figure 1D) and CFU were determined after 48 h of incubation with antibiotic. The lowest concentration that prevented increase in CFU compared to the value at 24 h, was defined as the biofilm MIC of the respective drug. Determination of biofilm MBC_90_, the concentration of drug that reduces viable numbers 10-fold compared to the CFU/peg at 24 h, were carried out the same way with increased concentrations of drug up to 100 μM.

### Survival and MBC_90_ Determination Under Nutrient Starvation

Exponentially growing Mab Bamboo precultures (section Growth in Aerated Nutrient-Rich Broth and MIC, MBC_90_ Determination) were spun down at 3200 × *g* for 10 min at 25°C and washed three times with phosphate buffered saline containing 0.025% Tyloxapol (Sigma-Aldrich T0307-10G, St. Louis, MO, United States) (PBS/Tyloxapol). Washed culture was then diluted with PBS/Tyloxapol to OD_600_ = 0.2 (1.8 × 10^7^ CFU/mL). One hundred milliliter of OD_600_ = 0.2 cultures were incubated in roller bottles (Corning 430195, Oneonta, NY, United States) at 37°C and 2 rpm. CFU were determined by plating. For determination of MBC_90_ against nutrient-starved non-replicating culture, 1 mL aliquots of 144 h old roller bottle cultures (Figure 1A) were transferred into 14 mL vented, round-bottom tubes (Thermo Fisher 150268, Rochester, NY, United States) and appropriate concentrations of drugs up to 100 μM were added. The tubes were incubated on a tilted rack at 37°C on an orbital shaker at 220 rpm for 48 h of incubation with antibiotics before plating and CFU enumeration.

### Survival and MBC_90_ Determination Under Anaerobic Conditions

Exponentially growing Mab Bamboo precultures (section Growth in Aerated Nutrient-Rich Broth and MIC, MBC_90_ Determination) were diluted with fresh 7H9 medium to OD_600_ = 0.02. 7 mL aliquots were transferred to 10 mL air-tight vacutainer tubes (BD 366430, Franklin Lakes, NJ, United States) containing elliptical stir bars (Radleys RR98096, Wood Dale, IL, United States). Tubes were incubated at 37°C on magnetic stirring platforms at 150 rpm. To monitor depletion of oxygen, methylene blue was added to cultures at a concentration of 1.5 μg/mL. The dye decolorized around day 5, indicating anaerobiosis. CFU were determined by plating. For determination of MBC_90_ against anaerobic non-replicating culture, appropriate concentrations of drugs up to 100 μM were added using a 23G needle (BD 305194, Franklin Lakes, NJ, United States) to minimize the re-introduction of air at 144 h (Figure 1B), i.e., after reaching anaerobiosis, and cultures were incubated for 48 h with antibiotics before plating and CFU enumeration.

Note: In the current work we use the term “persister assays” for the assays described under sections Growth as Biofilm and MIC, MBC_90_ Determination, Survival and MBC_90_ Determination Under Nutrient Starvation, and Survival and MBC_90_ Determination Under Anaerobic Conditions. By this we mean assays that grow bacterial cultures that show a shift in MIC and/or MBC_90_ to higher values when compared to the standard planktonic, aerated nutrient rich assay. Thus, these bacteria show “drug tolerance” or “phenotypic drug resistance.” For a detailed discussion (and various definitions) of these non-genetic resistance phenomena we refer to reviews by Gold and Nathan (Gold and Nathan, 2017) for mycobacteria and Balaban and colleagues (Balaban et al., 2019) for a consensus statement.

### Determination of Chromosome Equivalent Numbers

Genomic DNA of Mab Bamboo was extracted from bacterial culture samples following the Qiagen DNA extraction protocol and using the QIAamp 96 DNA Qiagen Kit (Sukumar et al., 2014). Samples were thawed and 100 μL was transferred to a 96 well plate (Qiagen 19585, Germantown, MD, United States) containing 180 μL of ASL buffer (Qiagen 19082, Germantown, MD, United States), 20 μL of proteinase K (Qiagen 19131, Germantown, MD, United States), 1 μL of reagent DX (Qiagen 19088, Germantown, MD, United States) and 250 μL of 0.1 mm zirconia-silica beads (MP Biomedicals 116540428, Solon, OH, United States) in each well. Samples were inactivated for 1 h at 80°C. The plate was centrifuged at 200 × *g* to remove foam and precipitate beads, then loaded in a QIAcube HT (Qiagen). Samples were mixed with 600 μL of 50% buffer AL (Qiagen 19075, Germantown, MD, United States) and 50% ethanol (Fisher Scientific A962-4, Fair Lawn, NJ, United States). 600 μL of supernatant from each well was transferred onto a 96 well plate silica column (Qiagen 28181, Germantown, MD, United States) and drawn through using a vacuum pump. DNA samples were cleaned with 3 consecutive wash steps: 600 μL of buffer AW1 (Qiagen 19081, Germantown, MD, United States), 600 μL of AW2 (Qiagen 19072, Germantown, MD, United States) and 600 μL of ethanol. DNA was then eluted with a total of 100 μL buffer AE (Qiagen 19077, Germantown, MD, United States). Mab Chromosome Equivalent (CEQ) was quantified using the previously described protocol (Munoz-Elias et al., 2005) with *erm*41 primer-probe combination [Primers: *5′-AAACCGTGAACGAAGGTGTC-3′* and *5′-TGGTATCCGCTCACTGATGA-3′* Probe: *5′-/56-FAM/CCGAATCCG/ZEN/GTGTTCGCTCA/3lABkFQ/-3′*] (Integrated DNA Technologies, Coralville, IA, United States) adapted from Lin et al. (2014). CEQ quantification was achieved by building standard curves using serial dilutions of whole Mab genome prepared from broth culture. Real time quantitative PCR (qRT-PCR) reactions were performed in duplicate at dilution 1x, 3x, and 9x (for a total of six q-PCR measures per sample) on the Mx3005P (Stratagene) using TaqMan Universal Master Mix II (Life Technologies 4440038, Carlsbad, CA, United States).

## Results

### *M. abscessus* Bamboo Survives Nutrient and Oxygen Starvation in a Non-replicating State and Can Grow as Biofilm

To determine whether Mab Bamboo is capable of surviving nutrient starvation, aerated pre-cultures growing exponentially in nutrient rich broth were transferred to PBS devoid of any nutrients and CFU over time were measured. Figure 1A shows that nutrient starved cultures stopped growth and maintained viability for 10 days, the duration of the experiment. This result shows that Mab can survive in saline lacking nutrients without significant loss of viability.

To determine whether Mab is capable of surviving oxygen deprivation, we transferred exponentially growing Mab pre-cultures into sealed containers (containing nutrient-rich broth), thus limiting the total amount of oxygen available. Figure 1B shows that Mab grows for 1 day and then enters stationary phase. Growth termination occurred earlier and at a lower cell density when compared to aerated unsealed (thus providing unlimited oxygen supply) cultures in the same medium (Figure 1C), presumably because the oxygen concentration fell below a level supporting growth. Indeed, when the oxygen indicator dye methylene blue was added to the sealed cultures, fading of the color (indicating microaerobic conditions) was observed at day four and decolorization (indicating anaerobiosis) was observed around day five (Figure 1B). Anaerobic cultures maintained viability at least until day 10, the duration of the experiment. This result shows that Mab can survive anaerobiosis without significant loss of viability.

Together, these results show that obligate aerobe and chemo-organo-heterotroph Mab is capable of surviving the absence of oxygen as well as nutrients for extended periods of time in an apparent non-replicating state.

**FIGURE 2:**
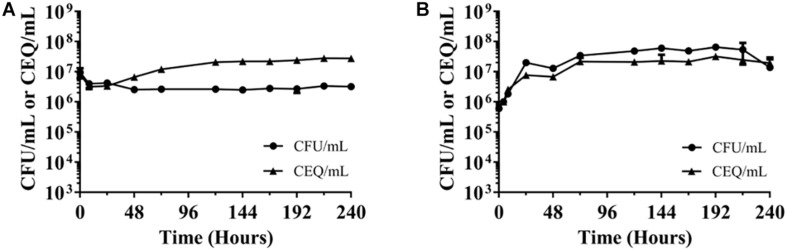
Chromosome equivalents (CEQ) vs. CFU of non-replicating *M. abscesuss* cultures. **(A)** Nutrient-starved cultures. **(B)** Oxygen-starved cultures. CEQ/mL vs. CFU/mL for *M. abscessus* grown in phosphate buffered saline (see Figure 1A) and in sealed containers containing nutrient-rich broth (Figure 1B) are shown. CEQ were enumerated by using quantitative real-time PCR. Note: There appears to be an increase of CEQ/mL relative to CFU/mL in **(A)** during the first 3 days. The reason for this apparent growth remains to be determined. The experiments were carried out at least two times independently in duplicate and mean values with standard deviations are shown.

The observed constant CFU/mL in the nutrient and oxygen-starved cultures could be due to a balance of bacterial death and “cryptic” growth or the bacteria may enter a state of “non-replicating persistence,” i.e., stop growing but maintaining viability. To differentiate between these two possibilities, chromosome equivalents per mL (CEQ/mL) over time were determined. If the observed constant CFU/mL is due to balanced death and growth, CEQ/mL of culture should increase over time due to the accumulation of the chromosomes from dead bacteria. If, however, the bacteria adopt a state of non-replicating persistence, CEQ/mL should be constant. Figure 2 shows that in both, nutrient-starved and oxygen-deprived conditions, CEQ/mL of culture did not change in a major way over time. This suggests that the bacteria under both culture conditions enter a state of non-replicating persistence, i.e., the bacteria largely stop replication and maintain viability under these adverse conditions.

To determine whether Mab is capable of growing as biofilm, aerated (planktonic) pre-cultures growing exponentially in nutrient rich broth were transferred into the wells of multi-titer plates containing nutrient broth. A surface for biofilm growth was provided by pegs – attached to the specialized lid of the multi-titer plates – that were inserted into the cultures present in the wells. Bacteria in the broth of the wells were allowed to attach to the surface of the pegs for 1 day after which the lids with the pegs were transferred to new micro-titer plates containing nutrient-rich medium without bacteria. Growth of bacteria attached to the surface of the pegs was determined by recovering the bacterial films attached to the surface of the pegs and CFU enumeration. Figure 1D shows that bacteria attached to the pegs and grew exponentially on this surface for 2 days before entering stationary phase. These results show that Mab is capable of growing as biofilm on this plastic surface.

Taken together, these experiments show that Mab Bamboo is able to survive nutrient and oxygen starvation in a non-replicating state and can grow as biofilms. The described culture models are reproducible and can thus be used for testing for activity of anti-bacterials against the different physiological forms of Mab generated under these culture conditions.

### Non-replicating and Biofilm-Growing *M. abscessus* Bamboo Displays Tolerance to Clinically Used Antibiotics

Before determining the effect of being in a non-replicating state or growing as biofilm on drug susceptibility we established a baseline by measuring the standard MIC and MBC_90_ for clinically used anti-Mab drugs against Mab growing under standard conditions. First, exponentially growing, aerated nutrient-rich broth, planktonic Mab cultures were treated with increasing concentrations of drug to determine the concentration that inhibited growth (measured by CFU enumeration). Table 1 shows that most drugs show potent MICs. Smaller than 5 μM MIC values were displayed by the macrolides clarithromycin, azithromycin, the aminoglycoside amikacin, the glycylcycline tigecycline, the beta-lactam imipenem, the oxazolidinone linezolid, the fluoroquinolone moxifloxacin and the riminophenazine clofazimine. Only the tetracycline minocycline and the beta-lactam cefoxitin showed non-favorable MICs in the range of 20–30 μM against Mab Bamboo. To evaluate bactericidal activity of anti-Mab drugs under these standard conditions MBC_90_ values were measured. Again, Mab cultures exponentially and planktonically growing in aerated, nutrient-rich broth were treated with increasing concentrations of drug (starting with the MICs of the drugs and up to a maximum of 100 μM) to determine the effect on viability of the cultures (measured by CFU enumeration). Only four of the drugs – amikacin, imipenem, cefoxitin, and moxifloxacin showed appreciable bactericidal activity with MBC_90_ values between 3 and 25 μM. For clarithromycin, azithromycin, minocycline, tigecycline, linezolid, and clofazimine – 100 μM, the highest concentration tested, resulted in less than a 10-fold reduction of viable counts. Thus, these antibiotics did not display any significant bactericidal activity. This result shows that about half of the commonly used drugs are not bactericidal against rapidly growing planktonic cultures.

Next, we determine the bactericidal activity of the same drugs against non-replicating cultures, either induced by nutrient or by oxygen starvation. Table 1 shows that all the standard growth assay non-bactericidal drugs were also non-bactericidal against the non-replicating bacterium. Importantly, the antibiotics that had shown bactericidal activity against growing culture lost this activity completely against non-replicating culture (MBC_90_ > 100 μM). These results show that the non-replicating state of Mab displays extreme tolerance against currently used “bactericidal” antibiotics. It is interesting to note that the non-bactericidal clofazimine (MBC_90_ in broth culture > 100 μM) gained some bactericidal activity against oxygen starved bacteria (MBC_90_ = 31 μM). The mechanism of action of this riminophenazine is not known and the reason for the gain of bactericidal activity against anaerobic non-replicating Mab remains to be determined.

Above, we determined the effect of Mab entering non-replicating states on the bactericidal activity of drugs. Next, we asked whether growth as biofilm, as opposed to growth as planktonic culture, affects the MIC and the MBC_90_ of drugs. Table 1 shows that biofilm MICs increased for most drugs around 3–4-fold. This result suggests that Mab growing as biofilm is less susceptible to growth inhibition when compared to Mab growing in suspension culture. The determination of MBC_90_ for drugs against growing biofilm shows that this trend also holds true for bactericidal activities. Amikacin, imipenem, cefoxitin and moxifloxacin, the drugs that showed appreciable bactericidal activities against exponentially growing planktonic bacteria showed 2–8-fold shifts to higher MBC_90_ values against exponentially growing biofilm bacteria. Interestingly, the “outlier” was again clofazimine, showing an MBC_90_ of 12.5 μM against biofilm-growing bacteria whereas it was not bactericidal in broth culture (MBC_90_ > 100μM).

Together, these results show that about half of the anti-Mab drugs are not bactericidal even under standard aerated, nutrient-rich culture conditions which allow the bacteria rapid growth. The few drugs that show bactericidal activity against growing planktonic cultures lose this activity completely against non-growing forms of the bacterium, whether induced by nutrient starvation or oxygen depletion. This shows that non-replicating Mab displays extreme resistance to killing by our clinically used drugs. Finally, we find that Mab growing as biofilm displays tolerance to growth inhibition when compared to Mab growing in suspension culture. Furthermore, biofilm-growing bacteria show reduced killing by drugs that display bactericidal activity against planktonically growing Mab.

### Non-replicating and Biofilm-Growing *M. abscessus* Bamboo Is Susceptible to Novel Membrane Function Targeting Agents

The new “persister” culture models we developed here are intended to be used as secondary whole cell assays in Mab drug discovery flowcharts to complement standard MIC and MBC_90_ profiling of new anti-mycobacterial agents. The mycobacterial membrane and its associated functions recently gained interest as target space (Chen et al., 2018). For instance, several diverse chemotypes were found to show attractive potency against *M. tuberculosis* and Mab by inhibiting the essential mycolic acid transporter MmpL3 (Li et al., 2018). To determine the attractiveness of this target for Mab “persister” states we tested a novel N-substituted indolemethylamine (IMA6, Supplementary Figure S1) in our assays. IMA6 was identified in a parallel drug discovery project on *M. tuberculosis* and shown to target MmpL3 in this pathogen (Dick and Go, unpublished). Data displayed in Table 1 confirm that the compound also shows attractive MIC and bactericidal activity against Mab growing under standard conditions. Interestingly, bactericidal activity of IMA6 was retained against nutrient-starved and oxygen-depleted non-replicating states, and against biofilm-growing Mab. These results suggest MmpL3 as an attractive “anti-persister” target. As a second example we tested a novel N^1^,N^3^-dialkyl substituted dioxonaphthoimidazolium, SA23 (Supplementary Figure S2), which we are currently characterizing for activity against *M. tuberculosis*. In *M. tuberculosis* SA23 appears to act via redox cycling involving the electron transport chain (Dick and Go, unpublished). Table 1 shows that this agent displayed attractive MIC and exerts bactericidal activity against both oxygen deprivation as well as nutrient starvation induced non-replicating Mab. These results suggest that the described “persister assays” can be employed for the identification of novel chemical entities displaying bactericidal activity against Mab “persisters.”

## Discussion

Mab lung disease is a disabling chronic condition (Ryan and Byrd, 2018). Currently used antibiotics are not effective in eradicating this infection and new, more efficacious drugs are needed. An important observation is that MIC, the standard pharmacodynamic parameter for preclinical *in vitro* compound prioritization, appears to be of only limited predictive value for clinical efficacy (Wu et al., 2018). Despite the availability of highly potent antibiotics in terms of MIC values, cure rates are poor. Based on our drug discovery experience in tuberculosis, a disease which shows similarities in terms of pathology, we proposed recently that the compound progression flowchart for Mab drug discovery projects should include *in vitro* “persister assays” (Wu et al., 2018). Rapidly growing aerated nutrient-rich planktonic cultures may not reflect the predominant physiology of the bacteria growing in patients. MIC and MBC_90_ values derived from this assay may thus be of limited value for the identification of efficacious drugs. “Persister assays” attempt to mimic *in vivo* “culture conditions” and corresponding pathophysiology of the bacteria. Here, we developed and used such “persister assays.” We show that Mab Bamboo, a recent clinical isolate we use in our Mab drug discovery projects (Yee et al., 2017), is capable of surviving extended periods of time without oxygen or nutrients by shifting to a non-replicating state. As Mab was shown to grow as biofilms in patients (Qvist et al., 2015; Fennelly et al., 2016), we also developed an *in vitro* biofilm model for this strain. We showed that – consistent with previous data – only a few of the clinically used drugs display bactericidal activity even in the standard aerated, nutrient-rich growth assay (Maurer et al., 2014). These few drugs lose their bactericidal activity when tested against non-replicating forms of the bacterium. Thus, non-replicating states of Mab turned out to be extremely resistant against killing by “bactericidal” drugs. These results confirm and extend previous work showing that Mab is capable of entering non-replicating but viable states and that these physiological forms of the bacterium are tolerant to killing (Miranda-CasoLuengo et al., 2016; Berube et al., 2018). That drugs which are bactericidal against growing mycobacteria lose this property against non-growing forms was previously also shown for *M. tuberculosis* (Lakshminarayana et al., 2015). The underlying mechanisms for the loss of bactericidal activity of anti-mycobacterials remain to be determined but appear to include reduced drug uptake associated with the non-growing stage (Sarathy et al., 2013). Most antibiotics, although showing attractive MIC against replicating planktonic bacteria, showed higher MIC values against biofilm-growing bacteria. Biofilm-growing bacteria showed also reduced susceptibility to the killing activity of “bactericidal” antibiotics. These results are consistent with and extend previous findings, demonstrating drug tolerance of Mab biofilms (Howard et al., 2006; Greendyke and Byrd, 2008; Clary et al., 2018; Hunt-Serracin et al., 2019).

Our results may, at least in part, explain the poor efficacy of our current drugs regarding the eradication of Mab infections in patients. Mab *in vivo* may present in similar non-replicating or biofilm-growing states and thus be resistant to growth inhibition and/or killing by our current drugs. The “persister assays” we describe here using the Bamboo strain can be used for Mab drug discovery to prioritize compounds that are active against these drug tolerant forms of the bacterium. The assays are simple, reproducible, and have a direct viability readout (CFU). The CFU readout means that the assays are labor intensive and therefore should only be applied as secondary whole cell assays. The potential application of these assays for the discovery of novel “anti-persister” compounds is supported by the results of testing novel agents that involve bacterial membrane related functions in their mechanism of action: agents targeting the mycolic acid transporter MmpL3 or that undergo redox cycling showed activity against “persister” forms of Mab.

We believe that the described assays are useful additions to the Mab drug discovery tool box and fill an important assay gap (Wu et al., 2018). In the related field of tuberculosis drug discovery similar (and additional) secondary assays are widely used (Dartois and Barry, 2013; Gold and Nathan, 2017). The predictive value of these assays for Mab drug discovery can only be validated once we have efficacy data of new drugs that showed bactericidal activity in an *in vitro* “persister assay.”

## Data Availability Statement

All datasets generated for this study are included in the article/Supplementary Material.

## Author Contributions

Y-KY carried out the experiments. NA carried out the chromosome number determinations. M-LG synthesized IMA6 and SA23. Y-KY, M-LG, and TD wrote the manuscript.

## Conflict of Interest

The authors declare that the research was conducted in the absence of any commercial or financial relationships that could be construed as a potential conflict of interest.

## Abbreviations

Mab: *Mycobacterium abscessus.*
