# Supplementary material for: Extreme Drug Tolerance of Mycobacterium abscessus “Persisters”
Source: Front Microbiol. 2020 Mar 4;11:359. doi: 10.3389/fmicb.2020.00359 (PMC7064438; doi:10.3389/fmicb.2020.00359)
Supplement: Supplementary file 1 [file Data_Sheet_1.PDF]

Supplemental material

Figure S1 Structure of IMA6

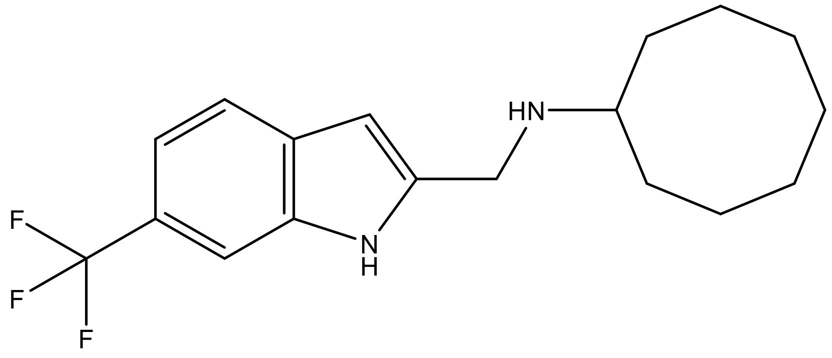

N-[(6-(Trifluoromethyl)-1*H*-indol-2-yl)methyl]cyclooctanamine

Figure S2 Structure of SA23

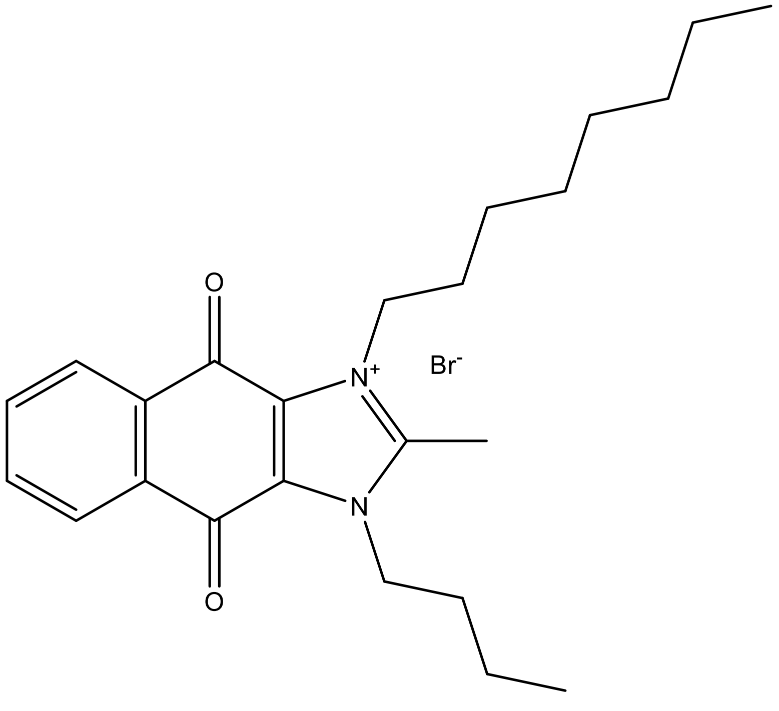

1-Butyl-2-methyl-3-octyl-4,9-dioxo-4,9-dihydro-1*H*-naphtho[2,3-*d*]imidazol-3-ium bromide
